# Sarcoma Immunotherapy

**DOI:** 10.3390/cancers3044139

**Published:** 2011-11-10

**Authors:** Launce G. Gouw, Kevin B. Jones, Sunil Sharma, R. Lor Randall

**Affiliations:** 1 Departments of Oncology, Huntsman Cancer Institute at the University of Utah, 2000 Circle of Hope, Salt Lake City, UT 84112, USA; E-Mail: sunil.sharmahciutah.edu (S.S.); 2 Departments of Orthopaedic Surgery, Huntsman Cancer Institute at the University of Utah, 2000 Circle of Hope, Salt Lake City, UT 84112, USA; E-Mails: kevin.jones@hci.utah.edu (K.B.J.); lor.randall@hci.utah.edu (R.L.R.)

**Keywords:** sarcoma, immunotherapy, immunosurveillance, vaccine

## Abstract

Much of our knowledge regarding cancer immunotherapy has been derived from sarcoma models. However, translation of preclinical findings to bedside success has been limited in this disease, though several intriguing clinical studies hint at the potential efficacy of this treatment modality. The rarity and heterogeneity of tumors of mesenchymal origin continues to be a challenge from a therapeutic standpoint. Nonetheless, sarcomas remain attractive targets for immunotherapy, as they can be characterized by specific epitopes, either from their mesenchymal origins or specific alterations in gene products. To date, standard vaccine trials have proven disappointing, likely due to mechanisms by which tumors equilibrate with and ultimately escape immune surveillance. More sophisticated approaches will likely require multimodal techniques, both by enhancing immunity, but also geared towards overcoming innate mechanisms of immunosuppression that favor tumorigenesis.

## 1. Introduction

Bone and soft tissue sarcomas compromise a rare and heterogeneous array of tumors derived from mesenchymal origins. Clinical behavior varies between subtypes, with a wide range of natural history, progression and susceptibility to therapy. The aggressive use of multimodal therapy has improved outcomes in a subset of sarcoma, but disease presenting with metastatic spread usually portends an extremely poor outcome. Immunotherapy targeted against sarcoma may open a new front in the battle against these malignancies, with utility in the neoadjuvant, adjuvant and metastatic settings.

## 2. Historical Precedents

Immune protection against tumors is a concept over a century old, waxing and waning in popularity over the decades. Importantly, one of the first germane observations relevant to this theory was predicated on an observation of a sarcoma patient. In 1891, Coley [1] noted a patient with small cell sarcoma cleared of disease following an erysipelas infection, proposing an anti-infectious mechanism with antitumor effects. He tested his hypothesis by inducing infections and later injecting heat-inactivated bacteria to stimulate an immune response with dramatic effects in some sarcoma patients [2]. In 1909, Paul Erlich [3] had expanded on earlier discussions regarding immunity [4] to encompass cancer as a target. Coley's results were difficult to replicate, and while the idea lost credence for a time, it was revived with mid-20^th^ century theories of “tumor surveillance” by Thomas [5] and Burnet [6,7]. Again initially discounted, this paradigm was eventually validated by a growing body of data, once again with sarcoma studies leading the way.

## 3. Pre- and Early Clinical Sarcoma Studies Contribute to a General Immunosurveillance Model

Following the observation that it was possible to induce immunity versus tumors previously felt to be non-immunogenic, provided the tumor cells were mutagenized with carcinogens [8], numerous subsequent studies using sarcoma mouse models extended our understanding of the immune system's ability to reject tumor cells. Preclinical studies with syngeneic mice injected with fibrosarcoma cells engineered to be insensitive to IFNγ demonstrated increased tumorigenicity versus their IFNγ-sensitive counterparts, inferring a role for this molecule in tumor cell immunogenicity, recognition and elimination [9]. These observations were fleshed out by the same investigators using IFNγ receptor knockout mice, [10] which were found to be more susceptible to sarcoma formation. Concurrent experiments noted perforin-deficient mice also demonstrated susceptibility to induced sarcomas [11]. With perforin's role as a key component of cytotoxic T and NK cell lymphocyte-dependent killing, this line of evidence underscored the key role of these cells in rejecting induced malignancies. Similarly, mice deficient in genes necessary for NK, T and B cell antigen receptor rearrangement were also found to develop sarcomas at a significantly greater rate than control animals [12]. Together, these findings revived the notion of immune system management of primary tumor development, with a central role for cytotoxic T-lymphocytes (CTLs) and their cytokines.

While these preclinical studies revitalized the idea of tumor immunosurveillance, evidence in the clinical realm kept in step with preclinical findings. A growing awareness of increased cancer susceptibility in immunocompromised patients was recognized, in both immunosuppressed transplant patients [13] as well as those harboring congenital or acquired immunodeficiencies [14,15]. A wide variety of tumor types have been recognized in several epidemiological studies of such patients, including sarcoma [16,17]. HHV 8-related Kaposi sarcoma is a model for viremia-associated tumors in immunocompromised hosts, but those without a known viral etiology have also been noted. In addition, the finding of individuals with CTL infiltration of tumors harboring a more favorable prognosis has been similarly suggestive of the crucial role the immune system plays in combating tumorogenesis [18].

The combined preclinical and clinical studies provide the basis of the “cancer immunoediting” paradigm. Three key phases of the current model are described: elimination, equilibrium and escape [19]. In the *elimination* phase, the innate and adaptive elements of immune system recognize and destroy nascent tumor cells. Some tumor cells survive and are “sculpted” by the immune system in a selection process to reach a degree of *equilibrium* [12,20] whereby they may persist, but not progress. Finally, a subset of these equilibrated tumor cells *escape* immune management by disabling immune recognition, often co-opting the system for growth, invasion and angiogenesis [21-26]. Further investigation of these phases are instrumental in understanding how the immune system works against cancer, how it can be subverted by tumor cells, and ultimately in harvesting the power of immunity in the search for therapeutic alternatives.

## 4. Vaccine Studies

Despite preclinical precedents, sarcoma immunotherapy remains poorly characterized in humans. Many sarcoma subtypes harbor attractive targets for immune-mediated strategies due to the presence of specific epitopes, either based on cell of origin or alterations of gene products, leading to ongoing enthusiasm for this technique. Specifically, up to a fourth of sarcomas are characterized by reproducible genetic changes, potentially providing tumor-specific immune recognition sites. Unfortunately, meaningful responses to vaccination in solid tumors has been disappointing, with “modern” efforts in sarcoma dating back some 40 years [27]. Vaccines targeting whole cells, lysates, proteins, and peptides specific to sarcoma subtypes (especially translocation-specific epitopes) have been tested to little avail, although several clinical studies are ongoing in the hopes of a breakthrough (Table 1).

The lack of efficacy seen by these sarcoma studies may partially be due to the lackluster response by T-cells responding to the vaccine. Often less than 1% of the CTLs with the desired T-cell receptor are detected in the peripheral circulation following vaccination trials, in contrast to the avid response of CTLs to viral epitopes (in the range of 20-50% or more). Attempts with costimulators such as saponin, KLH, or Freund's adjuvant have not improved response markedly, nor has addition of other immune stimulants such as GMCSF or IL-2. However, improving the yield of active CTLs *in vivo* is unlikely to be enough. Even in situations where as many as 30% of circulating CD8+ T-cells demonstrate anti-tumor reactivity, tumors can still thrive [28] The poor results of vaccine-only trials to date are likely due largely to the equilibrium and escape mechanisms sarcomas undergo in their evolution.

Key to the equilibrium and escape mechanisms are other players in the panoply of immune mediators. Regulatory CD4+ CD25+ T cells (Tregs), first recognized in the early 1990s, are becoming better characterized, acting to suppress autoimmunity and playing a protective role for tumors [41]. Inhibitory cell surface components on infiltrating lymphocytes, such as PD-1 [42] and CTLA-4 [43] have been determined, with their presence resulting in CTL suppression. Also, CD11b+ and Gr-1+ myeloid-derived suppressor cells (MDSCs) [44] and tumor associated macrophages (TAMs) [44,45] play additional functions in immune suppression and tumor promotion, acting within the tumor microenvironment. As it has become increasingly clear that standard approaches of generating a response to a sarcoma-specific epitope are insufficient to mount a significant clinical tumor response, it appears likely further manipulations are necessary.

Standard chemotherapeutics may be useful, as alkylating agents like cyclophosphamide or ifosfamide decrease Treg cells as part of their cytotoxic profile [46]. One may speculate that the efficacy of these agents in many sarcomas may be due, in part, to this action. Alkylators and other standard cytotoxics also destroy other suppressor cells such as MDSCs and TAMs, but have the undesired effect of indiscriminate destruction of desired CTLs. In order to circumvent the mediating influences of immune suppressors, newer agents targeting inhibitory cell surface components are being added to the arsenal of immunotherapeutics. The anti-CTLA4 agent ipilimumab is approved for melanoma immunotherapy and currently in clinical trials for patients with a variety of other solid tumors including various sarcoma subtypes (NCT00556881), although a trial with another anti-CTLA4 monoclonal antibody, MDX-010, in synovial sarcoma patients (NCT00140855) was closed due to poor accrual. Means to inhibit Tregs, MDSCs and TAMs are also being developed, acting by direct neutralization [47-50], or differentiation [51]. Human recombinant IL7 is a promising agent in this regard, as this cytokine appears to not only increase T-cell cycling and mass and subsequent T-cell receptor diversity, but concomitantly downregulates Treg activity [52,53].

## 5. T-Cell Approaches: Passive and Active

In addition to the above approaches, complementary strategies for vaccine-based immunotherapy that have demonstrated encouraging results in melanoma (and, more recently, prostate cancer [54]) continue to hold promise in sarcoma. They include adoptive or “passive” immunotherapy, with administration of CTLs selected *in vitro* for high affinity to tumor antigens that have undergone *ex vivo* expansion, free from negative selection pressure present during thymic development [55]. An advantage of this method when combined with standard chemotherapy is that it addresses the indiscriminate cytotoxicity of chemo as is seen with alkylators described above. Prior studies have laid the foundation for this approach in sarcoma with interesting results, [56] and a recent adoptive immunotherapy study by Robbins *et al* [36] has demonstrated promise in synovial sarcoma. In this small study, patients with tumors expressing the NY-ESO-1 antigen (expressed in 80% of synovial sarcomas) were recruited, along with NY-ESO-1 positive melanoma patients. Subjects were treated with a lymphodepleting chemotherapy regimen of cyclophosphamide and fludarabine followed by infusion of genetically modified autologous T lymphocytes designed to recognize a specific NY-ESO-1 epitope. Four of the six sarcoma patients (all of whom had progressed through multiple chemotherapeutic regimens), achieved a PR lasting from 5 to 18 months; 5/11 melanoma patients demonstrated clinical benefit, including two CRs. It should be noted that such treatments are not entirely “passive”, as the endogenous immune system is significantly modified by not only the adoptive transfer of T cells, but by lymphodepleting conditioning and post infusion immunomodulators given as part of most protocols.

Distinct from adoptive or “passive” techniques, “active” immunotherapy methodologies continue to garner attention. Clinical sarcoma investigators have long tried various schemes with immune activators and cytokine stimulators, some with intriguing results. The CCG/POG phase III intergroup trial 0133 enrolled 677 osteosarcoma patients from 1993-7, randomizing half the patients to arms where they received adjuvant chemotherapy with liposomal muramyl tripeptide phosphatidylethanolamine (L-MTP-PE) as a biological response modifier [57, 58]. L-MTP-PE was chosen for its utility as a monocyte/macrophage activator, with downstream activation of other components of the immune response. In the presence of this agent, risk of recurrence and death were decreased 25% and 30% respectively. Due to the 2 × 2 factorial design of the study, a potential interaction between L-MTP-PE and ifosfamide initially confounded the results. However, in followup analysis, a difference in disease-free survival (DFS) was noted, and although this result did not achieve significance, overall survival demonstrated a slight but significant improvement [59]. Unfortunately, for patients with metastatic disease, L-MTP-PE did not achieve a statistically significant improvement in outcome [60].

Dendritic cells (DCs) are distinct from other antigen-presenting cells in their ability to induce primary T-cell responses, and defined subsets of mature DCs may have direct antitumor activity as well [61]. Conversely, immature DCs can induce either suppressive TReg cells or unresponsive T-cells tilting the balance towards equilibrium or escape. A more technically complex “active” immunotherapeutic process is *ex vivo* expansion and maturation of antigen-presenting DCs pulsed with antigen, reintroducing these cells to the host as a form of a DC vaccine. DC vaccines have achieved a measure of success in other solid tumors and remain an active area of research in sarcoma. Although several DC-based vaccines continue to elicit interest in early phase sarcoma trials, they have thus far not panned out [40,62], though a current trial NCT00923351 is in process, utilizing a more thoroughly developed protocol with a more sophisticated DC scheme.

## 6. Immune Modulators and Targeting

Interferon is another “active” immunotherapeutic, an adjuvant effector presumed to act by myriad pathways, including directing macrophages towards cytotoxic antigen presenting phenotypes [63], connecting the innate and adaptive cellular immunity by enhancing the expression of tumor-associated antigens, promoting dendritic cell maturation and antigen presentation and promoting clonal expansion, survival and memory differentiation of T lymphocytes, especially the Th1 subset participating in cell-mediated immunity. Interferon has been investigated in osteosarcoma, both preclinically and clinically,with intriguing results [64-67]. Interferon therapy may also demonstrate limited utility in other subsets of sarcoma, such as desmoid tumors, epithelioid hemangioendothelioma, giant cell tumor of the bone and gastrointestinal stromal tumor (GIST), although this effect is primarily based on descriptive reports or case series, and would still be considered highly investigational in these settings. However, A recent phase II trial combining targeted therapy with interferon cytokine stimulation in patients with GIST has demonstrated interesting results [68]. The design utilized the action of the tyrosine kinase inhibitor imatinib to cause apoptosis and necrosis in sensitive GIST cells, followed by immunomodulation by peginterferon α-2b to stimulate the adaptive cellular immunity program. The advantage of imatinib over standard cytotoxics in this situation is not only its far superior efficacy against GIST, but its presumed ability to act without major destruction of the machinery necessary for an active immune response; indeed, imatinib appears to have inherent immune-modulatory properties, particularly in GIST [69,70]. Although the study was small and primarily hypothesis-generating, a more durable treatment response was noted in all patients compared to genotype-specific historical controls.

In yet another line of attack, immunotherapeutics may involve direct antibody recognition of sarcoma-specific epitopes, targeting the associated cells for destruction. Antibody-dependent cell-mediated toxicity approaches in sarcoma have yet to reach the success of the anti-CD20 antibody rituximab against CD-20 positive hematological malignancies. However, lymphoma immunoradiotherapeutics such as Y-90 labelled ibritumomab tiuxetan and I-131 labelled tositumomab may someday have analogues that are applicable to sarcoma. This is being investigated with the monoclonal antibody 8H9, which targets the 4Ig-B7-H3 epitope expressed in a variety of neuroectodermal, epithelial, and mesenchymal tissue, with high specificity to sarcoma, glioma and neuroblastoma [38]. This antibody has been radiolabelled with I-131 with success in a mouse model of rhabdomyosarcoma [71] and is moving forward with Phase I trials in humans (MSKCC protocol 09-090).

## 7. Conclusions

In the future, further immunomodulatory steps may be required for immunotherapy to achieve meaningful success against myriad sarcoma subtypes. T-lymphocytes, activated macrophages and monocytes, NK and mature dendritic cells and effector cytokines can be manipulated as described previously. Not only should attention be paid to enhancing immune elements, but also to defeating immunosuppressive elements to combat immune tolerization. Combined strategies could therefore also address such players as immature dendritic cells, MDSCs, co-opted TAMs and Treg cells and associated suppressor cytokines within the tumor microenvironment.

Much as the current state of the art in sarcoma treatment relies on integrated multidisciplinary efforts, it is very likely that such a combinatorial process will be necessary to maximize the promise of immunotherapy in this realm. In the future, as in the present, ultimate success will likely require input from multiple specialties dedicated to sarcoma. Primary tumor surgical resection/debulking and/or metastectomy combined with preparative chemotherapy and/or radiotherapy may well complement the efficacy of immunotherapeutics. Sarcoma remains an attractive immune target despite previous disappointments and as the process is more clearly elucidated, this procedure, once honed, will likely become an invaluable weapon in our therapeutic arsenal against a challenging and many-faced malignancy.

## Figures and Tables

**Table 1.** Antigen epitopes with potential utility in sarcoma immunotherapy. Unless otherwise specified, references are described in [29]. It should be noted that some of the proposed epitopes are based on wide ranging of preclinical evidence.

**Antigens**	**Sarcoma type**	**Selected references**

*Partial specificity:*		
XAGE-1	Ewing	
LIP1	all sarcoma	[30]
GM2	all sarcoma	[31,32]
GD2, GD3	all sarcoma	
PRAME	synovial, myxoid liposarcoma	
MAGE	synovial,leiomyosarcoma, osteosarcoma	[33]
GAGE	malignant fibrous histiocytoma	
WT-1	rhabdomyosarcoma	
FGR4	rhabdomyosarcoma	
ALK	rhabdomyosarcoma	[34,35]
NY-ESO-1	synovial	[36,37]
4Ig-B7-H3 (8H9 target)	all sarcoma	[38]
SSX2, SSX3	synovial	
Her2Neu	osteosarcoma	

*Sarcoma-specific fusion proteins:*		

SSX fusions	synovial	[39]
EWSR1 fusions	Ewing family	[40]
FOXO1 fusions	alveolar rhabdomyosarcoma	[40]
TLS-CHOP (FUS-DDIT3)	myxoid liposarcoma	
